# Supplementary material for: Global Distribution Prediction of Cyrtotrachelus buqueti Guer (Coleoptera: Curculionidae) Insights from the Optimised MaxEnt Model
Source: Insects. 2024 Sep 17;15(9):708. doi: 10.3390/insects15090708 (PMC11431845; doi:10.3390/insects15090708)
Supplement: Supplementary file 1 [file insects-15-00708-s001.zip › insects-3162695-supplementary.pdf]

Locations of ***Cyrtotrachelus buqueti***.

Longitude	Latitude	Sources
98.9	18.8	[1]
100.4	19.1	[1]
101.2	17.1	[1]
102.6041667	29.89583333	[2]
102.6458333	29.72916667	[2]
102.6458333	30.1875	[2]
102.6875	29.8125	[2]
102.7291667	29.6875	[2]
102.7291667	29.77083333	[2]
102.7291667	29.85416667	[2]
102.7291667	29.97916667	[2]
102.7708333	29.85416667	[2]
102.7708333	29.89583333	[2]
102.7708333	30.0625	[2]
102.7708333	30.1875	[2]
102.8125	29.72916667	[2]
102.8125	29.8125	[2]
102.8125	29.85416667	[2]
102.8125	29.89583333	[2]
102.8125	29.97916667	[2]
102.8125	30.02083333	[2]
102.8125	30.10416667	[2]
102.8125	30.14583333	[2]
102.8125	30.3125	[2]
102.8541667	29.77083333	[2]
102.8541667	29.8125	[2]
102.8541667	29.85416667	[2]
102.8541667	30.0625	[2]
102.8541667	30.10416667	[2]
102.8958333	29.72916667	[2]
102.8958333	29.8125	[2]
102.8958333	29.9375	[2]
102.8958333	29.97916667	[2]
102.8958333	30.02083333	[2]
102.9375	29.97916667	[2]
102.9375	30.0625	[2]
102.9375	30.14583333	[2]
102.9375	30.22916667	[2]
102.9375	30.27083333	[2]
102.9791667	29.77083333	[2]
102.9791667	30.0625	[2]

102.9791667	30.10416667	[2]
102.9791667	30.39583333	[2]
103.0208333	29.85416667	[2]
103.0208333	30.02083333	[2]
103.0208333	30.22916667	[2]
103.0625	29.89583333	[2]
103.0625	29.9375	[2]
103.0625	30.1875	[2]
103.0625	30.35416667	[2]
103.0625	30.60416667	[2]
103.1041667	29.72916667	[2]
103.1041667	29.8125	[2]
103.1458333	29.85416667	[2]
103.1458333	30.27083333	[2]
103.1458333	30.3125	[2]
103.1875	29.6875	[2]
103.1875	29.97916667	[2]
103.2291667	29.77083333	[2]
103.2291667	29.8125	[2]
103.2291667	29.9375	[2]
103.2291667	30.27083333	[2]
103.2291667	30.35416667	[2]
103.2708333	29.89583333	[2]
103.2708333	30.3125	[2]
103.2708333	30.4375	[2]
103.3125	28.89583333	[2]
103.3125	29.4375	[2]
103.3125	29.85416667	[2]
103.3125	29.9375	[2]
103.3125	30.02083333	[2]
103.3125	30.35416667	[2]
103.3125	30.4375	[2]
103.3125	30.5625	[2]
103.3541667	29.85416667	[2]
103.3541667	30.27083333	[2]
103.3541667	30.52083333	[2]
103.3958333	28.6875	[2]
103.3958333	29.47916667	[2]
103.3958333	29.8125	[2]
103.3958333	29.9375	[2]
103.3958333	30.02083333	[2]
103.3958333	30.0625	[2]
103.3958333	30.64583333	[2]
103.3958333	30.6875	[2]

103.4375	28.9375	[2]
103.4375	29.52083333	[2]
103.4375	29.97916667	[2]
103.4375	30.10416667	[2]
103.4375	30.1875	[2]
103.4375	30.39583333	[2]
103.4375	30.6875	[2]
103.4791667	29.60416667	[2]
103.4791667	29.85416667	[2]
103.4791667	30.10416667	[2]
103.4791667	30.14583333	[2]
103.5208333	28.64583333	[2]
103.5208333	28.72916667	[2]
103.5208333	28.8125	[2]
103.5208333	28.9375	[2]
103.5208333	29.02083333	[2]
103.5208333	29.9375	[2]
103.5208333	30.02083333	[2]
103.5208333	30.1875	[2]
103.5208333	30.22916667	[2]
103.5208333	30.27083333	[2]
103.5208333	30.60416667	[2]
103.5208333	30.6875	[2]
103.5625	28.77083333	[2]
103.5625	28.85416667	[2]
103.5625	29.52083333	[2]
103.5625	30.0625	[2]
103.6041667	28.89583333	[2]
103.6041667	28.97916667	[2]
103.6041667	29.0625	[2]
103.6041667	29.14583333	[2]
103.6041667	29.27083333	[2]
103.6041667	29.5625	[2]
103.6041667	30.02083333	[2]
103.6041667	30.10416667	[2]
103.6041667	30.14583333	[2]
103.6041667	30.27083333	[2]
103.6458333	28.6875	[2]
103.6458333	29.52083333	[2]
103.6458333	29.60416667	[2]
103.6458333	29.64583333	[2]
103.6458333	29.9375	[2]
103.6875	28.60416667	[2]
103.6875	28.8125	[2]

103.6875	28.9375	[2]
103.6875	29.02083333	[2]
103.6875	29.52083333	[2]
103.6875	29.5625	[2]
103.6875	29.64583333	[2]
103.6875	30.02083333	[2]
103.6875	30.14583333	[2]
103.7291667	28.6875	[2]
103.7291667	29.10416667	[2]
103.7291667	29.1875	[2]
103.7291667	29.22916667	[2]
103.7291667	29.52083333	[2]
103.7291667	29.60416667	[2]
103.7291667	29.6875	[2]
103.7291667	30.0625	[2]
103.7291667	30.1875	[2]
103.7291667	30.22916667	[2]
103.7291667	30.3125	[2]
103.7708333	28.5625	[2]
103.7708333	28.85416667	[2]
103.7708333	28.97916667	[2]
103.7708333	29.14583333	[2]
103.7708333	29.22916667	[2]
103.7708333	29.27083333	[2]
103.7708333	29.64583333	[2]
103.7708333	29.72916667	[2]
103.7708333	29.8125	[2]
103.7708333	29.9375	[2]
103.7708333	30.02083333	[2]
103.7708333	30.10416667	[2]
103.7708333	30.22916667	[2]
103.7708333	30.3125	[2]
103.8125	29.14583333	[2]
103.8125	29.1875	[2]
103.8125	29.27083333	[2]
103.8125	29.52083333	[2]
103.8125	29.5625	[2]
103.8125	29.60416667	[2]
103.8125	29.6875	[2]
103.8125	29.89583333	[2]
103.8125	29.97916667	[2]
103.8125	30.14583333	[2]
103.8125	30.27083333	[2]
103.8125	30.47916667	[2]

103.8541667	28.6875	[2]
103.8541667	28.85416667	[2]
103.8541667	29.02083333	[2]
103.8541667	29.0625	[2]
103.8541667	29.14583333	[2]
103.8541667	29.64583333	[2]
103.8541667	29.72916667	[2]
103.8541667	29.77083333	[2]
103.8541667	29.8125	[2]
103.8541667	29.85416667	[2]
103.8541667	29.9375	[2]
103.8541667	30.14583333	[2]
103.8541667	30.1875	[2]
103.8541667	30.3125	[2]
103.8541667	30.35416667	[2]
103.8541667	30.52083333	[2]
103.8541667	30.5625	[2]
103.8541667	30.60416667	[2]
103.8958333	28.9375	[2]
103.8958333	28.97916667	[2]
103.8958333	29.0625	[2]
103.8958333	29.22916667	[2]
103.8958333	29.27083333	[2]
103.8958333	29.52083333	[2]
103.8958333	29.5625	[2]
103.8958333	29.8125	[2]
103.8958333	29.89583333	[2]
103.8958333	30.02083333	[2]
103.8958333	30.27083333	[2]
103.9375	28.8125	[2]
103.9375	29.14583333	[2]
103.9375	29.27083333	[2]
103.9375	29.35416667	[2]
103.9375	29.5625	[2]
103.9375	29.60416667	[2]
103.9375	29.64583333	[2]
103.9375	29.72916667	[2]
103.9375	29.89583333	[2]
103.9375	29.9375	[2]
103.9375	29.97916667	[2]
103.9375	30.02083333	[2]
103.9375	30.0625	[2]
103.9375	30.14583333	[2]
103.9375	30.1875	[2]

103.9375	30.27083333	[2]
103.9375	30.3125	[2]
103.9375	30.47916667	[2]
103.9791667	28.64583333	[2]
103.9791667	29.0625	[2]
103.9791667	29.22916667	[2]
103.9791667	29.27083333	[2]
103.9791667	29.3125	[2]
103.9791667	30.35416667	[2]
103.9791667	30.39583333	[2]
103.9791667	30.47916667	[2]
104.1	30.4	[1]
104.0208333	28.89583333	[2]
104.0208333	28.97916667	[2]
104.0208333	29.02083333	[2]
104.0208333	29.0625	[2]
104.0208333	29.10416667	[2]
104.0208333	29.27083333	[2]
104.0208333	29.39583333	[2]
104.0208333	30.02083333	[2]
104.0208333	30.0625	[2]
104.0208333	31.4375	[2]
104.0625	28.72916667	[2]
104.0625	29.1875	[2]
104.0625	29.3125	[2]
104.0625	29.85416667	[2]
104.0625	29.89583333	[2]
104.0625	30.35416667	[2]
104.0625	30.4375	[2]
104.0625	31.27083333	[2]
104.1041667	28.85416667	[2]
104.1041667	28.97916667	[2]
104.1041667	29.10416667	[2]
104.1041667	29.22916667	[2]
104.1041667	29.27083333	[2]
104.1041667	29.35416667	[2]
104.1041667	29.89583333	[2]
104.1041667	30.27083333	[2]
104.1041667	31.3125	[2]
104.1041667	31.35416667	[2]
104.1041667	31.39583333	[2]
104.1458333	28.64583333	[2]
104.1458333	28.72916667	[2]
104.1458333	28.97916667	[2]

104.1458333	29.1875	[2]
104.1458333	29.3125	[2]
104.1458333	29.6875	[2]
104.1458333	30.10416667	[2]
104.1458333	30.3125	[2]
104.1458333	30.52083333	[2]
104.1875	29.52083333	[2]
104.1875	29.5625	[2]
104.1875	29.60416667	[2]
104.1875	30.22916667	[2]
104.1875	31.4375	[2]
104.2291667	28.89583333	[2]
104.2708333	28.77083333	[2]
104.2708333	29.27083333	[2]
104.3125	28.35416667	[2]
104.3125	28.85416667	[2]
104.3125	29.72916667	[2]
104.3541667	28.52083333	[2]
104.3541667	29.27083333	[2]
104.3958333	29.39583333	[2]
104.3958333	29.47916667	[2]
104.4375	28.89583333	[2]
104.4375	28.97916667	[2]
104.4791667	29.60416667	[2]
104.5208333	28.9375	[2]
104.5208333	29.35416667	[2]
104.6458333	29.22916667	[2]
104.6458333	29.27083333	[2]
104.6458333	29.60416667	[2]
104.6875	29.22916667	[2]
104.7708333	29.0625	[2]
104.7708333	29.14583333	[2]
104.8125	29.0625	[2]
104.8958333	28.35416667	[2]
104.8958333	29.35416667	[2]
104.9375	28.35416667	[2]
104.9375	28.4375	[2]
104.9375	29.27083333	[2]
104.9791667	28.3125	[2]
105.0208333	28.5625	[2]
105.0208333	28.64583333	[2]
105.0208333	29.02083333	[2]
105.0208333	29.14583333	[2]
105.0625	28.52083333	[2]

105.0625	28.6875	[2]
105.1041667	28.47916667	[2]
105.1041667	29.0625	[2]
105.1041667	29.22916667	[2]
105.1458333	28.0625	[2]
105.1458333	28.64583333	[2]
105.1458333	28.6875	[2]
105.1458333	29.02083333	[2]
105.1875	28.52083333	[2]
105.1875	29.10416667	[2]
105.1875	29.35416667	[2]
105.2291667	28.60416667	[2]
105.2291667	28.97916667	[2]
105.2291667	29.27083333	[2]
105.2708333	28.6875	[2]
105.2708333	29.47916667	[2]
105.3125	28.52083333	[2]
105.3541667	28.60416667	[2]
105.3541667	28.6875	[2]
105.3541667	28.72916667	[2]
105.4375	28.6875	[2]
105.4375	28.77083333	[2]
105.4791667	28.5625	[2]
105.4791667	28.72916667	[2]
105.5208333	28.6875	[2]
105.5625	28.77083333	[2]
105.6875	28.52083333	[2]
105.8	28.3	[1]
106.1041667	29.10416667	[2]
106.6041667	29.6	[1]
106.6041667	30.72916667	[2]
106.6458333	30.22916667	[2]
106.6458333	30.60416667	[2]
106.6458333	30.64583333	[2]
106.6458333	30.8125	[2]
106.6875	30.1875	[2]
106.6875	30.35416667	[2]
106.6875	30.39583333	[2]
106.6875	30.6875	[2]
106.6875	30.77083333	[2]
106.6875	30.8125	[2]
106.7291667	30.22916667	[2]
106.7291667	30.27083333	[2]
106.7291667	30.64583333	[2]

106.7291667	30.77083333	[2]
106.7708333	30.3125	[2]
106.7708333	30.35416667	[2]
106.8125	30.27083333	[2]
106.8541667	30.3125	[2]
106.8958333	30.0625	[2]
106.8958333	30.14583333	[2]
106.8958333	30.22916667	[2]
106.9375	30.3125	[2]
106.9375	30.39583333	[2]
106.9791667	30.10416667	[2]
107.0208333	30.14583333	[2]
107.0208333	30.47916667	[2]
107.0625	30.10416667	[2]
107.1041667	30.1875	[2]
107.1041667	30.3125	[2]
107.1041667	30.4375	[2]
107.1458333	30.1875	[2]
107.1875	30.22916667	[2]
107.7708333	30.89583333	[2]
107.7708333	31.14583333	[2]
107.8125	31.22916667	[2]
107.8958333	30.89583333	[2]
107.8958333	31.0625	[2]
107.9375	31.02083333	[2]

[1] GBIF.org (31 July 2024) GBIF Occurrence Download https://doi.org/10.15468/dl.msv3qz, accessed on 31 July 2024 and Peng, Yaqin (2024).
[2] Wang J ,Wang F ,Wang R , et al.Modeling the effects of bioclimatic characteristics and distribution on the occurrence of Cyrtotrachelus buqueti in the Sichuan Basin[J].Global Ecology and Conservation,2019,17e00540-e00540.
